# Estimating Inhalation Exposure Resulting from Evaporation of Volatile Multicomponent Mixtures Using Different Modelling Approaches

**DOI:** 10.3390/ijerph19041957

**Published:** 2022-02-10

**Authors:** Martin Tischer, Michael Roitzsch

**Affiliations:** BAuA: Federal Institute for Occupational Safety and Health, Unit “Exposure Scenarios”, Friedrich-Henkel-Weg 1-25, 44149 Dortmund, Germany; roitzsch.michael@baua.bund.de

**Keywords:** volatile multicomponent mixtures, evaporation models, mass balance, near-/far field, continuous application, extended Euler algorithm, backpressure, activity coefficients, occupational exposure, biocidal products

## Abstract

In many professional and industrial settings, liquid multicomponent mixtures are used as solvents, additives, coatings, biocidal products, etc. Since, in all of these examples, hazardous liquids can evaporate in the form of vapours, for risk assessments it is important to know the amount of chemicals in the surrounding air. Although several models are available in legal contexts, the current implementations seem to be unable to correctly simulate concentration changes that actually occur in volatile mixtures and in particular in thin films. In this research, the estimation of evaporation rates is based on models that take into account non-ideal behaviour of components in liquids and backpressure effects as well. The corresponding system of differential equations is solved numerically using an extended Euler algorithm that is based on a discretisation of time and space. Regarding air dispersion of volatile components, the model builds upon one-box and two-box mass balance models, because there is some evidence that these models, when selected and applied appropriately, can predict occupational exposures with sufficient precision. That way, numerical solutions for a wide variety of exposure scenarios with instantaneous and continuous/intermittent application, even considering “moving worker situations”, can be obtained. A number of example calculations have been carried out on scenarios where binary aqueous solutions of hydrogen peroxide or glutaraldehyde are applied as a biocidal product to surfaces by wiping. The results reveal that backpressure effects caused by large emission sources as well as deviations from liquid-phase ideality can influence the shape of the concentration time curves significantly. The results also provide some evidence that near-/far-field models should be used to avoid underestimation of exposure in large rooms when small/medium areas are applied. However, the near-field/far-field model should not be used to estimate peak exposure assuming instantaneous application, because then the models tend to overestimate peak exposure significantly. Although the example calculations are restricted to aqueous binary mixtures, the proposed approach is general and can be used for arbitrary liquid multicomponent mixtures, as long as backpressure effects and liquid-phase non-idealities are addressed adequately.

## 1. Introduction

For occupational risk assessments, often evaporation of volatile multicomponent mixtures is of interest. In many processes, liquid mixtures are used as coatings, solvents, fuels, additives, etc. Models such as ConsExpo [1] and ART [2] are frequently used to estimate exposure for the risk assessment carried out under the biocides regulation [3] or REACH [4]. However, although these models are basically able to handle mixtures, the current implementations seem to be unable to correctly simulate concentration changes that actually occur in volatile mixtures and in particular in thin films [5]. A common task is, for instance, the application of a disinfectant which involves spreading the product onto a surface, e.g., by mopping or wiping. This may lead to incorrect assessments of the exposure situations especially if mixtures consist of components with significantly different volatilities. That is, the models cannot depict local differences in concentrations and quantities that occur within a larger surface during continuous application due to evaporation that has already progressed to different degrees.

Hence, we think that characterizing pollutant sources in terms of their emissions is a basic parameter of indoor air systems. The chemicals released into the air together with the room geometry and ventilation conditions produce the air concentrations in a room that result in worker exposure. If one assumes that a substance enters a room (by whatever means), the time course of the substance concentration can be described on the basis of a mass balance. The balance states that the temporal change of a substance concentration in a balance room can be described by the sums of the substance flows entering and leaving the balance room. Thus, two essential elements of any mass balance model are the emission rate and the room ventilation; both must be defined for the model.

The models employed today in occupational exposure assessment are typically based on some simple assumptions about airflow and contaminant transport pattern. This research builds up on well-mixed room (one-box) and near-field/far-field (two-box) models, because there is some evidence [6,7] that these models, when selected and applied appropriately, can predict occupational exposures with sufficient precision to drive appropriate exposure and risk management decision making. While well-mixed room models assume a uniform substance concentration throughout one room, near-field/far-field models are the simplest mathematical models to reflect spatial variability in exposure because they divide the environment into two or more contiguous, exclusive volumes. Since workers typically move in their working environment, spatial variability should not be neglected in model building especially when room volumes are large. Additionally, the pattern of application can also determine the spatial position of the emission source, e.g., when continuously spreading products, such as mopping with a disinfectant.

Regarding the application pattern, there are two principal modes addressed in this study. First, the product is applied instantaneously to a constant surface area, and second, the product is applied continuously/intermittently (possibly by a moving worker) so that the evaporation surface area is increasing over time. The theoretical concept of a new approach using mass balance models will be described in the following sections in more detail. Special situations where unintended spills play a role are not further addressed.

## 2. Materials and Methods

### 2.1. Estimating Evaporation Rates of Liquid Mixtures

A number of models have been proposed for the evaporation of substances from open surfaces, each of which makes different assumptions and simplifications. The simplest approach regards a pure substance as applied instantaneously and then evaporated from a surface area that remains the same size over time. It is clear that instantaneous application is an ideal that will rarely occur in practice. However, the assumption “instantaneous” is regarded as reasonable if the application time is small in comparison to the whole evaporation time.

More importantly, exposure to vapours at workplaces often originates from liquid mixtures. Mixtures and especially the influence of liquid phase non-ideality on evaporation have gained relatively little recognition, however. One model that performed well under a variety of conditions [8] and that is able to predict evaporation rates of liquid mixtures was developed by Gmehling and Weidlich [9,10], s. Equation (2). According to the two-film theory, in this model it is assumed that evaporation is driven by the difference between the partial vapour pressure of an individual component i, which can be derived from the equilibrium vapour pressure by Raoult’s law, and its vapour pressure in the room air *p*_i,room_, which in this context is often referred to as “backpressure”. Raoult’s law relates the partial vapour pressure of compound i to its saturation vapour pressure *p*_i_^*^ and its molar fraction *x*_i_, which is calculated by

(1) $$x_{i,\mathrm{liq}}=\frac{n_{\mathrm{liq},i\;}}{\sum_{j}n_{\mathrm{liq},i}}$$

The evaporation rate of substance i, $\frac{dn_{\mathrm{evap},i}}{dt}$ , is proportional to this pressure difference and depends on the surface area A of the product and the mass transfer coefficient *ß*_i_. The activity coefficient *γ*_i_ ${,}_{\mathrm{liq}}$ is used to take non-ideality into account (vide infra).

(2) $$\frac{dn_{\mathrm{evap},i}}{dt}=\frac{A\cdot \beta_{i}}{R\cdot T}\cdot \left(x_{liq,i}\cdot \gamma_{\mathrm{liq},i}\cdot p_{i}^{*}-p_{\mathrm{room},i}\right)$$

The vapour pressure in the room air can be related to its concentration using the ideal gas law.

(3) $$p_{i,\mathrm{room}}=\frac{n_{\mathrm{room},i}\cdot R\cdot T}{V_{\mathrm{room}}}=\frac{c_{\mathrm{room},i}\cdot R\cdot T}{M_{i}}$$

If the vapour pressure in the room air is higher than the partial vapour pressure, Equation (2) will result in a negative evaporation rate. This can be suppressed by introducing the Heaviside operator *H*.

(4) $$H\left(x\right)=\{\begin{array}{c} 0,\;\;\;\;\;\;\;\;\;0<x\\ 1,\;\;\;\;\;\;\;\;\;0\geq x \end{array}$$

Finally, the modified Gmehling–Weidlich equation describing the evaporation rate can then be written as:

(5) $$\frac{dn_{\mathrm{evap},i}}{dt}=\frac{A\cdot \beta_{i}}{R\cdot T}\cdot \left(x_{\mathrm{liq},i}\cdot \gamma_{\mathrm{liq},i}\cdot p_{i}^{*}-p_{\mathrm{room},i}\right)\cdot H\left(x_{\mathrm{liq},i}\cdot \gamma_{\mathrm{liq},i}\cdot p_{i}^{*}-p_{\mathrm{room},i}\right)$$

The mass transfer coefficient *ß*_i_ is a function of the diffusivity *D*_i_ of the substance in the air and of the air flow *v*_air_ over the product surface. Parameter notation and corresponding symbols and units are listed in Table 1.

(6) $$\beta_{i}=0.011\cdot \frac{v_{\mathrm{air}}^{0.96}\cdot \;D_{i}^{0.19}{}^{\;}}{\nu^{0.14}\cdot X^{0.04}}$$

The values of the molecular diffusion coefficient *D*_i_ and substance vapour pressure are available for many substances in chemical property reference manuals. For chemicals for which the molecular diffusion data and vapour pressure are not available, there are some estimation methods, e.g., Equation (10) in McCready and Fontaine [11].

The parameter exponents in Equations (2) and (6) were fitted based on experiments carried out by Gmehling and Weidlich [9,10] for different solvents released from mixtures at air velocities from 0.2 to about 0.7 m/s and low air concentrations. Due to the low air velocities, it can be assumed that these equations best represent the laminar flow conditions. Slow, laminar flows have a different influence on mass transfer than fast turbulent air flows, as the latter additionally leads to turbulent back mixing above the evaporation surface. Hence, for fast turbulent airflows, other models may be more appropriate [12]. It has to be noted that the model of Gmehling and Weidlich assumes isothermal evaporation at a constant room temperature *T*, which means that cooling effects are neglected. The more volatile the substances are, cooling effects may play a significant role, leading to a reduction in vapour pressure and consequently in air concentrations. From this, it follows that Equation (2) may tend to overestimate exposure slightly. However, overestimation can often be accepted since conservative estimates are more justifiable from an occupational safety and health view.

As mentioned above, in the Gmehling–Weidlich equation, liquid phase non-ideality is corrected for by use of the activity coefficient *γ*_i,liq_, which is concentration dependent. The importance of taking deviations from Raoult’s law into account even for moderately non-ideal mixtures has been demonstrated by various authors (see, e.g., [12]). Activity coefficients for a wide range of mixtures can be estimated using the group-contribution concept of the UNIFAC method that was introduced by Fredenslund, Jones, and Prausnitz [13] and was further investigated by Gmehling [14]. The group-contribution concept is based on the idea that physicochemical properties of organic molecules can be represented reasonably well by segmenting a molecule into different “functional” groups and considering specific properties of these groups. This leads to the description of a liquid mixture as a “solution of groups” (instead of a solution of molecules). This concept has been proven useful; it is often a good approximation for the real behaviour of organic mixtures and aqueous–organic mixtures. However, there are also limitations to the group-contribution approach, especially when applied to inorganic–organic mixtures. In order to overcome these limitations, Zuend et al. [15,16] have developed the thermodynamic model AIOMFAC that is designed for the calculation of activity coefficients of different chemical species in inorganic–organic mixtures. The AIOMFAC model has been developed and tested against hundreds of experimental datasets and the model has been shown to be useful, reasonably accurate, and practical for the calculation of activity coefficients in complex multicomponent mixtures [17,18].

Another useful approach for predicting activity coefficients involves the use of an equation of state to represent the behaviour of the gas phase and an excess Gibbs energy model to represent the behaviour of the liquid phase. In particular, the Margules equation [19], the van Laar equation [20], the Wilson equation [21], the NRTL equation [22], and the UNIQUAC equation [23] have found widespread usage. From a computational point of view, these methods correlate the activity coefficients *γ*_i_ of a compound i with their mole fractions *x*_i_ in the liquid phase by a separate function, i.e., γ_i_ = *f*(*x*_i_). Basically, they can be used to estimate activity coefficients for all mixture compositions. In practice, however, there is often a lack of the needed model parameters, which prevents the models from being used consistently. Therefore, different activity coefficient models were used in each case in this study (s. chapter results), depending on the particular composition of the mixture and the data available on model parameters.

In some cases, the non-ideal effect is less pronounced, and it may be argued that non-ideal solution calculations are not necessary for certain mixtures. In practice, however, only people with in-depth thermodynamic knowledge can predict when mixtures do not deviate significantly from Raoult’s law. Hence, it is recommended to consider the relevance of non-ideal behaviour prior to any assessment.

For exposure assessments, usually the concentration of a substance i in the room air, *C*_room,i_, denoted in mass per volume, is of interest. For setting up and discussing the mass balance equations, however, it is easiest to observe the molar amounts, for example the molar amount of substance i in the room air, *n*_room,i_. Considering that the transition between concentrations and molar amounts using the relation $C=\frac{n\cdot M}{V}$ is rather simple, the subsequent presentation of the computational methods will focus on the molar amounts.

### 2.2. Well-Mixed Room Model for Instantaneous Application (WMR_inst)

In the instantaneous application scenario, a certain molar amount *n*_init,A,i_ of the substance i (as a component of the liquid mixture) is applied initially to a certain surface area *A*. Then, evaporation starts, and owing to differences in volatility of individual components, usually the composition, and therefore the evaporation rate of the liquid mixture, changes over time. During evaporation, the concentration of the evaporated substances is highest near the source and forms a concentration gradient throughout the room. However, for simplification, often an even distribution is assumed. This approach, which is known as a well-mixed room model [24], is reasonable when room volumes are small and when the room is well mixed due to either natural or induced air currents, resulting in nearly equal concentration levels throughout the room. Assuming ideal conditions, the molar amount of compound i in the room air, *n*_room,i_, is a function of the evaporation rate $\frac{dn_{\mathrm{evap},i}}{dt}$ (s. Equation (7)) and the loss caused by the ventilation rate *Q*_vent_ expressed in (m^3^/s). These relationships can be expressed as follows:

(7) $$\frac{dn_{\mathrm{room},i}}{dt}=\frac{dn_{\mathrm{evap},i}}{dt}-Q_{\mathrm{vent}}\cdot \frac{n_{\mathrm{room},i}}{V_{\mathrm{room}}}+Q_{\mathrm{vent}}\cdot \frac{C_{\mathrm{vent},i}}{M_{i}}$$

The term *C*_vent,i_ describes the concentration of compound i in the supply air. As mentioned above, the desired air concentration at time *t* can be easily derived from this equation using the ideal gas law. This results in:

(8) $$\frac{dC_{\mathrm{room},i}}{dt}=\frac{M_{i}}{V_{\mathrm{room}}}\cdot \frac{dn_{\mathrm{evap},i}}{dt}-\frac{Q_{\mathrm{vent}}}{V_{\mathrm{room}}}\cdot C_{\mathrm{room},i}+\frac{Q_{\mathrm{vent}}}{V_{\mathrm{room}}}\cdot C_{\mathrm{vent},i}$$

The loss of compound i occurring from the liquid layer due to evaporation is considered by Equation (9), which is here just the negative of the evaporation rate (see Equation (5)).

(9) $$\frac{dn_{\mathrm{liq},i}}{dt}=-\frac{dn_{\mathrm{evap},i}}{dt}$$

Based on these assumptions, the complete mass balance of this system can be described by a set of two time-varying differential equations for each of the involved compounds. It is important to note that all differential equations describing the various components in the liquid are coupled via the molar fraction *x*_liq,i_ in Equation (5). As a consequence, the evaporation of all (major) volatile compounds from the liquid layer has to be taken into account. This leads to the question of whether there is a closed-form analytical expression for the integration describing the time varying amounts *n*_room,i_ of each substance in the room air. Unfortunately, this finding process, using symbolic mathematics programs such as Mathematica [25], was not successful. Hence, numerical solutions of the simultaneous differential equations for instantaneous application were obtained using a fourth-order Runge–Kutta method [26] (s. chapter results). For the rather simple Runge–Kutta method, the number of iteration steps must be chosen. During our work, a number in the range of 10,000 steps has proven to be a good compromise between computing speed and accuracy.

### 2.3. Near-Field/Far-Field Model for Instantaneous Application (NF/FF_inst)

The obvious drawback to the well-mixed room model is that concentration gradients between the source and the rest of the room are ignored. This may result in underestimation of the exposure, because in most scenarios, the exposed person is located next to the source. One way to account for the concentration gradients that occur in a room is to divide the airspace into two or more “zones”. This has the advantage of accounting for the “positional variability” in concentration by using relatively simple mathematical approaches.

For the application pattern “instantaneous”, a two-compartment box model with an emission source in the near field was used. Here, it is assumed that an initial molar amount *n*_init,A,i_ of substance i (as a component of the liquid mixture) is instantaneously applied to a certain surface area *A* which is completely located in the near field. In addition to the approach discussed above, this model comprises terms for the volumes of the near field (*V*_nf_) and the far field (*V*_ff_); the quantity of the interzonal air flowing from the near to the far field and vice versa is given by *Q*_nf/ff_, which is assumed to be due to natural convection. The distribution of the substance i in the near field (*n*_nf,i_) and the far field (*n*_ff,i_) is assumed to be homogenous within the respective volumes. The evaporation term changes slightly compared to Equation (5) in that now the backpressure inside the near field, *p*_nf,i_ has to be considered:

(10) $$\frac{dn_{\mathrm{evap},\mathrm{nf},i}}{dt}=\frac{A\cdot \beta_{i}}{R\cdot T}\cdot \left(x_{\mathrm{liq},i}\cdot \gamma_{\mathrm{liq},i}\cdot p_{i}^{*}-p_{\mathrm{nf},i}\right)\cdot H\left(x_{\mathrm{liq},i}\cdot \gamma_{\mathrm{liq},i}\cdot p_{i}^{*}-p_{\mathrm{nf},i}\right)$$

The mass balance then comprises three simultaneous first-order differential equations for each component. The first expresses the concentration changes in the near field resulting from evaporation from the liquid into the near field and the air exchange between near and far field. The second equation describes the concentration changes in the far field due to exchange with the near field and the ventilation of the room (which is expected to occur inside the far field). As before, the last term on the right-hand side of Equation (12) represents the mass flow of component i by the incoming airflow *Q*_vent_. It has to be noted that for simplification, this mass flow is assumed to be zero in all numerical calculations discussed in this paper (which is probably not the case in practice). The third equation quantifies the changes within the liquid layer.

(11) $$\frac{dn_{\mathrm{nf},i}}{dt}=\frac{dn_{\mathrm{evap},\mathrm{nf},i}}{dt}-Q_{\mathrm{nf}/\mathrm{ff}}\cdot \frac{n_{\mathrm{nf},i}}{V_{\mathrm{nf}}}+Q_{\mathrm{nf}/\mathrm{ff}}\cdot \frac{n_{\mathrm{ff},i}}{V_{\mathrm{ff}}}$$

(12) $$\frac{dn_{\mathrm{ff},i}}{dt}=Q_{\mathrm{nf}/\mathrm{ff}}\cdot \frac{n_{\mathrm{nf},i}}{V_{\mathrm{nf}}}-Q_{\mathrm{nf}/\mathrm{ff}}\cdot \frac{n_{\mathrm{ff},i}}{V_{\mathrm{ff}}}-Q_{\mathrm{vent}}\cdot \frac{n_{\mathrm{ff},i}}{V_{\mathrm{ff}}}+Q_{\mathrm{vent}}\cdot \frac{c_{\mathrm{vent},i}}{M_{i}}$$

(13) $$\frac{dn_{\mathrm{liq},i}}{dt}=-\frac{dn_{\mathrm{evap},\mathrm{nf},i}}{dt}$$

**Figure 1:**
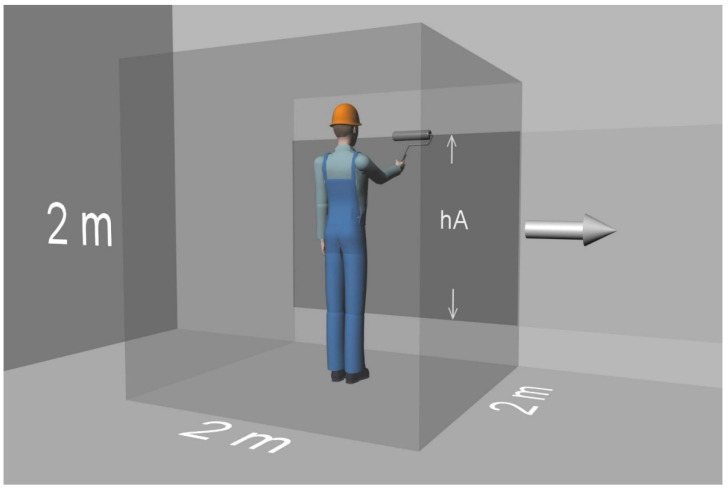
Concept of the two-zone model.

Regarding the most appropriate volume/location or geometry for the near field, so far, no consensus has emerged. We use an approach that is slightly modified in comparison to Cherrie and Schneider [27], who viewed the near field as a (2 × 2 × 2) m^3^ cube centred on the workers head. That is, one side face of the cube is 4 m^2^ and its volume is 8 m^3^. Unlike Cherrie and Schneider, we assume that the near field is centred on the workers body and thus includes the entire worker and the emission sources within reach of the worker (this is typically the case when liquid mixtures are manually applied to surfaces). In other words, we view the near field as encompassing the worker and an emission source area of 4 m^2^ at maximum (s. Figure 1). With this approach it is also possible to let the worker move in and out of the emission source area. Here, it is assumed that the total applied area *A* is a horizontal rectangle with arbitrary width and a height *h*_A_ that can be 2 m at maximum (s. Figure 1), but the same approach would be applicable to treatment of the floor for, e.g., mopping with a disinfectant.

The only model parameter which is essentially unknown is the interzonal airflow *Q*_nf/ff_. Following the approach of Cherrie [28] and Kulmala [29] to estimate *Q*_nf/ff_, it is assumed that there is a minimum convective airflow arising from the person’s body heat. Higher airflows are possible when air is moved through the near field from cross drafts, etc. For that reason, Cherrie has suggested interzonal airflows for three conditions: minimal likely convective airflow (3 m^3^/min), maximal convective airflow plus bulk air movement through the near field at 0.1 m/s (30 m^3^/min), and an intermediate value (10 m^3^/min). For the model calculation presented in this study, the intermediate value of 10 m^3^/min has been used (s. chapter results). Solutions for scenarios with instantaneous application (Equations (11)–(13)) were obtained numerically (s. chapter results).

### 2.4. Well-Mixed Room Model for Continuous Application (WMR_cont)

Although continuous application of volatile mixtures is quite common in practice, little attention has been given to this situation so far from a modelling perspective. Evans [30] developed continuous source terms for one- and two-compartment systems that break down the application into a sequence of differential area elements. Each area element has an emission profile assuming constant or exponentially decreasing emission rates. Hence, temporal and spatial differences in the emission rates of the area element can be represented. As time progresses during the application, the ensemble effect of the differential areas produces a “macroscopic” emission rate, which, in the absence of interaction, will be the sum of the microscopic rates. It must be emphasized that the approach of Evans [30] assumes that there is no feedback or “backpressure” effect from the room air, which would tend to suppress the microscopic emission rates as the room concentration increases. This kind of effect is certainly possible and relevant for large evaporating sources indoors and therefore regarded as a limitation.

Another approach that is able to model continuous application of volatile products is integrated into the evaporation model of ConsExpo [1] when choosing the release area mode “Increasing”. This model is based on a mass balance for the well mixed room. The mass transfer coefficient is estimated using alternatively Langmuir’s [31] or Thibodeaux’s expression [32]; or, in more recent versions, an empirically derived default value of 10 m/h is recommended. The model can take into account the increase in amount and surface area due to application of the product, thereby assuming a constant concentration of the mixture components and a constant film thickness over the surface. However, this assumption should be regarded as a limitation, because usually the composition and in consequence the evaporation of a liquid mixture in the surface layer changes over time and space, owing to differences in volatility of individual components.

To overcome these limitations, we suggest an approach that takes into account the backpressure effect and spatial and temporal differences of the evaporation rate. It has to be noted that the mathematical formalism using the Heaviside operator, as proposed by Evans [30], was not successful when backpressure plays a relevant role. Initially following Evans, we break down a certain area *A* into a sequence of *N*_ΔA_ small area elements ΔA, which are numbered consecutively using the index l. We assume that fractions of the product are applied instantaneously, element by element, where the applied amount reflects the work rate of the worker. However, in principle, this approach can also take arbitrary values for the applied amount, or even zero to reflect periods where the worker stops the application (e.g., breaks, post application phase).

The size of ΔA = *A/N*_ΔA_ determines the spatial resolution of the proposed approach. We then define the temporal resolution that is needed to describe the time-dependent evaporation from each area element appropriately. This is achieved by dividing the overall exposure time *t*_expo_ into *N*_t_ time steps so that Δ*t* = *t*_expo_/*N*_t_. Basically, the time Δ*t*_Δ__A_ needed to apply the product to one area element ΔA can be the same as Δ*t*, so that each time step involves the complete application to one area element. However, for diminishing calculation time, it may also be useful to reduce the spatial resolution by allowing Δ*t*_Δ__A_ to adopt integer multiples *IM* of Δ*t*, that is Δ*t*_Δ__A_ = Δ*t*·*IM*, ΔA = (*A/N*_t_)·*IM* and *N*_t_ *=* *N*_ΔA_·*IM*. For simplification, however, in the further discussion we will use *IM* = 1 until mentioned otherwise. How the temporal and spatial resolution determined by the size of *N*_t_ and *IM,* respectively, influences the numerical precision of this approach is addressed to some extent in the results chapter by some example calculations.

**Figure 2:**
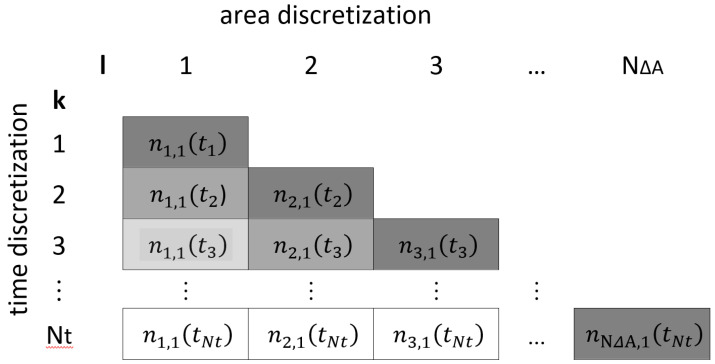
Discretisation approach for compound *I* = 1 and *IM* = 1.

Within the proposed procedure, we apply the initial molar amount n_liq,l,i,init_ of component i to an area element l and once Δ*t*_ΔA_ = Δ*t·IM* seconds have passed, we begin application to the next element l + 1. While this is crude for a small number of time steps, the approximation becomes much more reasonable when the time interval Δ*t* and the size of each area element ΔA decreases accordingly, approaching a quasi-continuous application. This discretisation approach is illustrated in Figure 2 for *IM* = 1 (that is Δ*t*_ΔA_ = Δ*t*). For each time step k and each area element l the molar amount of component i at the beginning of time step k is represented by $n_{1,t}\left(t_{k}\right)$ . For instance, $n_{2,1}\left(t_{3}\right)$ means the molar amount of component 1 on area element 2 at the beginning of time step 3. The diagonal elements (that is, k = l) of the matrix in Figure 2 represent the initial molar amount $n_{init,1,t}\left(t_{k}\right)$ of component i applied to area element l at the actual time *t*_k=l_ *= Δt ∙ (k − 1)*. The temporal iteration process starts with time step k = 1 and proceeds successively for each area element l to the maximum number of time steps *N*_t_. If *IM > 1,* the area size ΔA and the application time Δ*t*_ΔA_ of an area element needs to be adopted accordingly. Once some product is applied, the further course of the amount is determined by evaporation of the components i, which is again described by the Gmehling–Weidlich Equation (14), but now separately for each area element l and time step k using the results $n_{liq,1,t}\left(t_{k-1}\right)$ of the previous time step k − 1 as initial conditions.

$$k\in \mathbb{N},\;k=1,\;2\ldots \;{Nt}_{;}\;l\in \mathbb{N},\;l=1,\;2\ldots N_{\Delta A}$$

(14) $$\frac{dn_{\mathrm{evap},l,i}\left(t_{k}\right)}{dt}=\Delta A\cdot \beta_{i}\cdot \left(\frac{x_{\mathrm{liq},l,i}\left(t_{k}{}_{\;}\right)\cdot \gamma_{\mathrm{liq},l,i}\left(t_{k}{}_{\;}\right)\cdot p_{i}^{*}}{R\cdot T}-\frac{C_{\mathrm{room},i}\left(t_{k}\right)}{M_{i}}\right)\cdot H\left(\frac{x_{\mathrm{liq},l,i}\left(t_{k}{}_{\;}\right)\cdot \gamma_{\mathrm{liq},l,i}\left(t_{k}{}_{\;}\right)\cdot p_{i}^{*}}{R\cdot T}-\frac{C_{\mathrm{room},i}\left(t_{k}\right)}{M_{i}}\right)$$

It is assumed that there is no mass flow between neighbouring surface elements, but there is in the gas phase, which can be justified because the diffusion in gases is much faster (≈10^4^) than in liquids. This means that in this approach for each compound and each area element, individual evaporation rates will be computed, but only one rate in a common air space will be considered for a given point in time.

Hence, the average evaporation rate $dn_{\mathrm{evap},i}\left(t_{k}{}_{\;}\right)/dt$ over all area elements during the interval Δ *t* at time step k is approximated by the sum over all “active” area elements *N*_ΔA,k_ = *ceil*(*k/IM*)* *≤* N*_ΔA_ at time step k, where *ceil*(*x*) denotes the ceiling function, which gives the smallest integer greater than or equal to *x*). We can further assume that during sufficiently small time intervals Δ*t*, the evaporation rate remains almost constant.

This allows us to switch from differential terms to differences, i.e., $\frac{dn_{\mathrm{evap},i}\left(t_{k}{}_{\;}\right)}{dt}≅\frac{\Delta n_{\mathrm{evap},i}\left(t_{k}{}_{\;}\right)}{\Delta t}$ , and consequently:

(15) $$\frac{dn_{\mathrm{evap},i}\left(t_{k}{}_{\;}\right)}{dt}≅\sum_{l=1}^{N_{\Delta A,k}\;}\frac{n_{\mathrm{liq},l,i}\left(t_{k}{}_{\;}\right)-n_{\mathrm{liq},l,i}\left(t_{k+1}\right)}{\Delta t}$$

Since the molar fraction as well as the activity coefficients are concentration dependent, they have to be calculated individually for each area element and each time increment. Assuming the well-mixed room conditions, the temporal change of the air concentration $\frac{dC_{\mathrm{room},i}\left(t_{k}\right)}{dt}$ of component i at time step k is then approximated by Equation (16).

(16) $$\frac{dC_{\mathrm{room},i}\left(t_{k}\right)}{dt}=\frac{M_{i}}{V}\frac{dn_{\mathrm{evap},i}\left(t_{k}{}_{\;}\right)}{dt}-\frac{Q}{V}\cdot C_{\mathrm{room},i}\left(t_{k}{}_{\;}\right)+Q_{\mathrm{vent}}\cdot C_{\mathrm{vent},i}\left(t_{k}\right)$$

It is obvious that this approach will easily result in a massive number of differential equations when using a large number of area elements, which slows down the numerical solution with the Runge–Kutta method (or other methods such as DoPri-5). Since a very high numerical precision is not required in occupational exposure modelling, we therefore use the simplest method for numerical integration of differential equations, the Euler method [26]. Starting with the initial value *y*(*t* = 0), the Euler method calculates the absolute change of the target variable during this first interval Δ*y*(*t* = 0) by multiplying the slope *dy*(*t*)/*dt* at this point with the length Δ*t* of this interval. It then adds this value to the initial value of *y*(*t* = 0) to estimate the start value of the next interval and iterates this process until the last interval, i.e., *y*(t + Δ*t*) = *y*(*t*) + *dy*/*dt* · Δ*t*.

Although not required by the Euler method, for simplification we here use a constant time interval Δ*t* for the iteration steps. For an area element l and time step k, the iteration formulas of the Euler method for Equations (14)–(16) are then written:

(17) $$n_{\mathrm{liq},l,i}\left(t_{k+1}\right)=n_{\mathrm{liq},l,i}\left(t_{k}\right)-\Delta t\;\frac{dn_{\mathrm{liq},l,i}\left(t_{k}\right)}{dt}$$

(18) $$C_{\mathrm{room},i}\left(t_{k+1}\right)=C_{\mathrm{room},i}\left(t_{k}\right)+\Delta t\;\frac{dC_{\mathrm{room},i}\left(t_{k}\right)}{dt}$$

Since all time increments k have the same duration Δ*t*, they can simply be converted into an actual time *t*_k_ by:

(19) $$t_{k}=k\cdot \Delta t$$

Despite its flexibility, this extended Euler method can be programmed straight forward, essentially using two nested next loops. In Appendix A.1, a piece of pseudocode is given that is intended to illustrate how the iteration algorithm works for a binary mixture assuming continuous application under well-mixed room conditions.

The iteration process over time starts with initial values $n_{\mathrm{liq},l,i,\mathrm{init}}$ for each new area element added. In practical applications, the initial value can be constant or can change over time. For intermittent application, which is quite common in practice, using the Heaviside operator *H* we propose the following expression for $n_{\mathrm{liq},l,i,\mathrm{init}}$ :

(20) $$n_{\mathrm{liq},l,i,\mathrm{init}}=\overset{N_{\mathrm{AC}}}{\underset{m=1}{\sum }}\frac{SC_{i,m}\cdot r\cdot \Delta t\cdot IM}{M_{i}}\left(H\left(IM\cdot \Delta t\cdot \left(l-1\right)-t_{a,m}\right)-H\left(IM\cdot \Delta t\cdot \left(l-1\right)-t_{b,m}\right)\right)$$

$SC_{i,m}$ is the initial surface coverage in kg/m^2^ of component i for an application cycle m and *r* the coverage velocity in m^2^/s. The initial surface coverage $SC_{i,m}$ can take arbitrary positive values and can be calculated from the coverage of the product $SC_{P,m}$ over the mass fraction *w*_i_ of component i ( $SC_{i,m}=SC_{P,m}\cdot w_{i})$ . The term *t*_a,m_ is the starting time and *t*_b,m_ the end time of an application cycle m and *N*_AC_ denotes the total number of application cycles. Please note that in the case of intermittent application, the initial surface coverage can be zero as well, which means that no product is applied to the overall surface area *A*. This is, for instance, the case when the worker stops wiping, painting, etc., without leaving the room. A corresponding example for intermittent application is given in the results chapter.

### 2.5. Near-Field/Far-Field Models for Continuous Application (NF/FF_cont and NF/FF_mov)

The mass balance model described above can provide reasonable estimates when room volumes are small and the air is indeed well mixed. For large rooms, however, gradients of the airborne concentration can be quite high, especially due to temporal and spatial changes in the composition of the applied liquid mixture and due to incomplete air mixing. In addition, workers often move in large rooms, e.g., when manually applying disinfectants, paints, etc., to surfaces, leading to additional positional variability and in consequence possibly to misleading estimates of the airborne concentration. We therefore propose a near-field/far-field model that is able to address these scenarios to some extent. As before, this method employs a direct numerical solution based on the Euler method. As described in the chapter on near-field/far-field models for instantaneous application, we assume the near-field to be centred on the workers body and thus including the entire worker and the emission sources within reach of the worker. In this way, we can model situations in which the worker and the corresponding near field are stationary (that is, the worker can only move within the near-field limits), as well as situations in which the worker and the corresponding near field are moving. In this setup, evaporation occurs into the near field from area elements located close to the worker, but at the same time more distant area elements may evaporate directly into the far field. Thus, we now need two different equations (see Equations (21) and (22)) to describe the evaporation rate of component i for area element l, depending on whether the element is located in the near or far field. The question then arises of which area element l evaporates into the near field and which into the far field during a time increment k. The size of the surface area *A*_nf_ in the near field is here a key parameter as in our example, we assume a near field with dimensions of 2 × 2 × 2 m^3^, assuming a rectangular horizontal (e.g., floor) or vertical (e.g., wall) surface just fitting into this near field, i.e., 2 × 2 m^2^, which seems reasonable.

(21) $$\begin{array}{c} \frac{dn_{\mathrm{evap},\mathrm{nf},l,i}\left(t_{k}\right)}{dt}=\Delta A\cdot \beta_{i}\;\cdot \left(\;\frac{x_{\mathrm{liq},l,i}\left(t_{k}\right)\cdot \gamma_{\mathrm{liq},l,i}\left(t_{k}\right)\cdot p_{i}^{*}}{R\cdot \;T}-\frac{C_{\mathrm{nf},i}\left(t_{k}\right)}{M_{i}}\right)\\ \;\;\;\;\;\cdot H\left(\frac{x_{\mathrm{liq},l,i}\left(t_{k}\right)\cdot \gamma_{\mathrm{liq},l,i}\left(t_{k}\right)\cdot p_{i}^{*}}{R\cdot \;T}-\frac{C_{\mathrm{nf},i}\left(t_{k}\right)}{M_{i}}\right) \end{array}$$

(22) $$\begin{array}{c} \frac{dn_{\mathrm{evap},\mathrm{ff},l,i}\left(t_{k}\right)}{dt}=\Delta A\cdot \beta_{i}\;\cdot \left(\;\frac{x_{\mathrm{liq},l,i}\left(t_{k}\right)\cdot \gamma_{\mathrm{liq},l,i}\left(t_{k}\right)\cdot p_{i}^{*}}{R\cdot \;T}-\frac{C_{\mathrm{ff},i}\left(t_{k}\right)}{M_{i}}\right)\\ \;\;\;\;\;\cdot \left(\frac{x_{\mathrm{liq},l,i}\left(t_{k}\right)\cdot \gamma_{\mathrm{liq},l,i}\left(t_{k}\right)\cdot p_{i}^{*}}{R\cdot \;T}-\frac{C_{\mathrm{ff},i}^{\;}\left(t_{k}\right)}{M_{i}}\right) \end{array}$$

In contrast to the well-mixed room model the mass balance now can take emission sources in the near and the far field into account. Assuming a stationary near field, the average evaporation rate over the interval Δ *t* at time-step k is approximated by the sum of all active area elements in the near field *N*_ΔA,nf,k_ = *ceil*(*k/IM*)* *≤* N*_ΔA,nf_ and in the far field *N*_ΔA,ff,k_ = *ceil*(*k/IM*)** ≤ *N*_ΔA,ff_, respectively

(23) $$\frac{dn_{\mathrm{evap},\mathrm{nf},i}\left(t_{k}{}_{\;}\right)}{\Delta t}≅\sum_{l=1}^{N_{\Delta A,\mathrm{nf},k}}\frac{n_{\mathrm{liq},\mathrm{nf},l,i}\left(t_{k}{}_{\;}\right)-n_{\mathrm{liq},\mathrm{nf},l,i}\left(t_{k+1}\right)}{\Delta t}$$

(24) $$\frac{dn_{\mathrm{evap},\mathrm{ff},i}\left(t_{k}{}_{\;}\right)}{\Delta t}≅\sum_{l=1}^{N_{\Delta A,\mathrm{ff},k}}\frac{n_{\mathrm{liq},\mathrm{ff},l,i}\left(t_{k}{}_{\;}\right)-n_{\mathrm{liq},\mathrm{ff},l,i}\left(t_{k+1}\right)}{\Delta t}$$

The changing amounts of component i in the air of a stationary near field around the worker and in the far field at time step k are then approximated by Equations (25) and (26), respectively:

(25) $$\frac{dC_{\mathrm{nf},i}\left(t_{k}\right)}{dt}≅\frac{M_{i}}{V_{\mathrm{nf}}}\frac{dn_{\mathrm{evap},\mathrm{nf},i}\left(t_{k}{}_{\;}\right)}{dt}-\frac{Q_{\mathrm{nf}/\mathrm{ff}}}{V_{\mathrm{nf}}}\cdot C_{\mathrm{nf},i}\left(t_{k}\right)+\frac{Q_{\mathrm{nf}/\mathrm{ff}}}{V_{\mathrm{nf}}}\cdot C_{\mathrm{ff},i}\left(t_{k}\right)$$

(26) $$\frac{dC_{\mathrm{ff},i}\left(t_{k}\right)}{dt}≅\frac{M_{i}}{V_{\mathrm{ff}}}\frac{\Delta d_{\mathrm{evap},\mathrm{ff},i}\left(t_{k}{}_{\;}\right)}{dt}-\frac{Q_{\mathrm{nf}/\mathrm{ff}}+Q_{\mathrm{vent}}}{V_{\mathrm{ff}}}\cdot C_{\mathrm{ff},i}\left(t_{k}\right)+\frac{Q_{\mathrm{nf}/\mathrm{ff}}}{V_{\mathrm{ff}}}\cdot C_{\mathrm{nf},i}\left(t_{k}\right)$$

As the search for an analytical solution to this sequence of differential equations was not successful, we propose a numerical approximation using the Euler method again. With k∈ℕ,  k = 1, 2… *N*_t_*;* $l\in \mathbb{N},\;l=1,\;2..N_{\Delta A}$ , the iterative formulas are written as:

(27) $$n_{\mathrm{liq},\mathrm{nf},l,i}\left(t_{k+1}\right)=n_{\mathrm{liq},\mathrm{nf},l,i}\left(t_{k}\right)-\Delta t\cdot \frac{dn_{\mathrm{liq},\mathrm{nf},l,i}\left(t_{k}\right)}{dt}\;$$

(28) $$n_{\mathrm{liq},\mathrm{ff},l,i}\left(t_{k+1}\right)=n_{\mathrm{liq},\mathrm{ff},l,i}\left(t_{k}\right)-\Delta t\cdot \frac{dn_{\mathrm{liq},\mathrm{ff},l,i}\left(t_{k}\right)}{dt}$$

(29) $$C_{\mathrm{nf},i}\left(t_{k+1}\right)=C_{\mathrm{nf},i}\left(t_{k}\right)+\Delta t\cdot \frac{dC_{\mathrm{nf},i}\left(t_{k}\right)}{dt}$$

(30) $$C_{\mathrm{ff},i}\left(t_{k+1}\right)=C_{\mathrm{ff},i}\left(t_{k}\right)+\Delta t\cdot \frac{dC_{\mathrm{ff},i}\left(t_{k}\right)}{dt}$$

This iterative approach is quite flexible and allows us to model a variety of scenarios by choosing different initial conditions. We propose two scenarios that may be of practical relevance. In the case of a stationary near field, the product is applied continuously only within the reach of the worker. That is, the entire applied area must not exceed 4 m^2^, which is the side face of the near-field cube. Hence, no product is applied from the worker located in the stationary near field to the far field. With Equation (20), for intermittent applications, the user needs to specify the surface coverage $SC_{i,m}^{\;}$ and start/end times *t*_a,m_ and *t*_b,m_ of component i for each application cycle m, the total exposure time *t*_expo_ and the number of iteration steps *N*_t_ and *IM* to define the initially applied microscopic molar amounts. It should be noted that in the case of a stationary near field, continuous product application to the far field (by another worker) can be simulated as well. For reasons of brevity, however, we refrain from exemplifying.

The second scenario allows a moving near field (NF/FF_mov) and hence may be indicated if the worker needs to move when treating large surfaces/rooms. Here, it is assumed that the total applied area *A* is a horizontal rectangle with height *h*_A_, which can be 2 m at maximum (s. Figure 1). In the first phase, the product is applied by the worker continuously in the near-field area as long as the width of the applied area is less than 2 m. In this phase, the near field is assumed as stationary, and no product is applied to the far field. In the subsequent phase, the worker (and with him the near field) starts moving and continues applying the product until the entire rectangular area A is covered. That is, the worker and the corresponding near field can move out of the already applied area. This moves more and more applied near-field areas into the far field. Taking into account the height *h*_A_ of the applied area, the coverage velocity *r*, the width of the near field of 2 m and the exposure time *t*_expo_, the number of area elements in the moving near field $N_{\Delta A,\mathrm{nf}}$ can be calculated using the following expression:

(31) $$N_{\Delta A,\mathrm{nf}}\;=\frac{h_{A}\cdot 2\cdot N_{t}}{r\cdot t_{\mathrm{expo}}}$$

Whether a near-field area element starts emitting to the far field is decided by the criteria $N_{\Delta A,\mathrm{nf}}=k-l$ . In Appendix A.2, a piece of pseudocode is given that illustrates in more detail how the iteration algorithm works. It should be noted that the moving near-field algorithm assumes *IM* = 1 to keep spatial resolution at maximum.

## 3. Results

**Figure 3:**
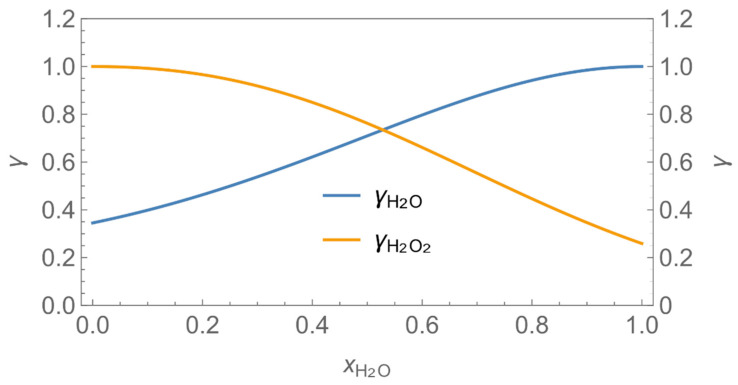
Activity coefficient of H_2_O_2_ in aqueous solutions.

**Figure 4:**
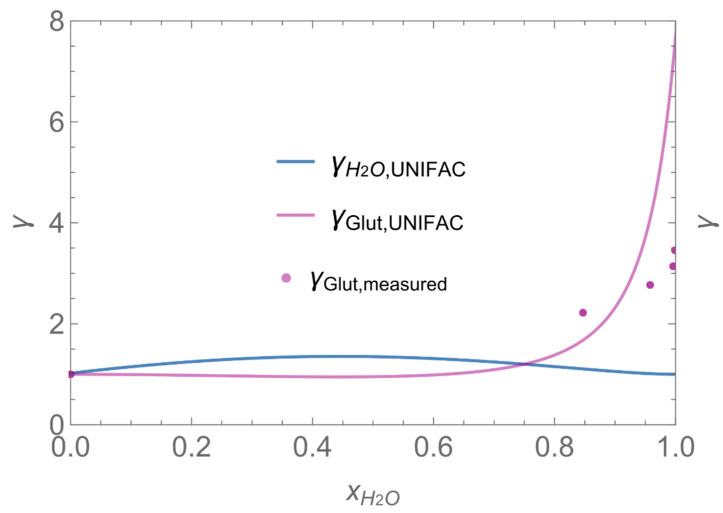
Activity coefficient of glutaraldehyde in aqueous solutions.

In order to demonstrate the advantages and drawbacks of the different model approaches, a number of example calculations have been carried out. Since the number of possible scenarios is endless, we decided to focus on scenarios where binary aqueous solutions of H_2_O_2_ or glutaraldehyde are applied as a biocidal product to surfaces by wiping, mopping, etc. In addition to the substance-specific saturation vapour pressures (p*H_2_O, p*H_2_O_2_, p*_glutarald_.), which have been looked up from the literature [33,34], the activity coefficients as a function of the mole fraction are required for the model calculations. While the activity coefficients for H_2_O_2_ and H_2_O have been estimated using the equation of Schumb et al. [35], which is based on measured data, the UNIFAC method was used to estimate the activity coefficient of glutaraldehyde in aqueous systems (s. Figure 3 and Figure 4). Figure 4 reveals that UNIFAC reflects the nonlinear behaviour of glutaraldehyde in aqueous mixtures fairly, although the estimated partial vapour pressures deviate from measured values to some extent. The initial mass fraction *w_i_* of these example substances is assumed to be 1% w/w, the initial surface coverage of the product *SC*_P_ = 0.1 kg/m^2^ and the application velocity *r* = 1 m^2^/min. Further substance-specific parameters are the mass transfer coefficients *β*_H2O_*, β*_H2O2_*, β*_glutarald_. that were calculated according to Equation (6). Values for the diffusion coefficient of the substance in air *D*_air_ and the kinematic viscosity of air *ν*_air_ were looked up from the literature [32] or estimated using the method presented by McCready and Fontaine [11], which is based on the work originally presented in Perry and Chilton [36]. The air velocity *v* and the interzonal ventilation rate *Q*_NF/FF_ as well as the air exchange *ACPH* of the room are assumed to be in a medium range. All numerical values of these input parameters are kept constant for all scenarios (s. Table 2).

In order to identify and illustrate the strengths and limitations of the modelling approach, several input parameters were varied when calculating the example scenarios. In addition to the size of the applied surface area and the application pattern, for some scenarios the influence of the backpressure as well as the influence of activity coefficients were considered (s. Table 3). It should be noted that the total amount applied is higher for the larger surfaces because the surface coverage per m^2^ is kept constant in all scenarios. In Table 4, the concentration time diagrams are depicted for the various scenarios for the systems H_2_O/H_2_O_2_ and H_2_O/glutaraldehyde. Please note that the left-hand ordinate uses different scales then the right-hand ordinate.

Assuming instantaneous application (s. Table 3), scenarios № 1 and № 2 reveal that the exposure level strongly depends on the size of the applied area and the amount. In addition, backpressure can influence the time course of release of H_2_O_2_ and glutaraldehyde significantly. Basically, there is a time shift to be expected in the release of both substances because both substances are less volatile than water, which causes water to evaporate first. However, as the comparison between № 1 and № 2 shows, it should be highlighted that this delay is enhanced if there are larger surfaces and amounts involved, potentially leading to higher evaporation rates and consequently more pronounced backpressure, which in turn delays evaporation and decreases the peak concentration. While the backpressure has a significant influence on the shape of the time course, the influence of the activity coefficients is not so pronounced in our examples, but it should be noted that for other mixtures, activity coefficients can deviate substantially from 1, making the influence of non-ideality much more pronounced. The concentration vs. time diagrams related to scenario 3 also reveal that the activity coefficients provoke different effects for H_2_O_2_ and glutaraldehyde. While the release of H_2_O_2_ is delayed at the beginning, glutaraldehyde starts to evaporate earlier to some extent. This is due to the fact that activity coefficients of H_2_O_2_ are below 1 (s. Figure 3) while activity coefficients for glutaraldehyde are significantly greater than 1 (s. Figure 4) for low concentrations. Thus, in the mixture, the effective vapour pressure of glutaraldehyde is greater than that of H_2_O_2_, although the saturation vapour pressure of pure glutaraldehyde is lower than that of H_2_O_2_. This effect can be observed if products are applied instantaneously (scenario № 3) and continuously (scenario № 4) as well. However, in contrast to instantaneous application, the concentration time curves are significantly broader with lower peak concentrations if products are applied continuously.

As demonstrated above, backpressure and activity coefficients can influence the shape of the concentration time curves significantly. In addition to these substance-specific parameters, concentration gradients between the source and the rest of the room can play an important role. This may result in underestimation of the exposure, especially in large rooms. The concentration time curves of scenario № 5 illustrate this effect, taking into account near-field/far-field modelling as well as backpressure and activity coefficients. While for instantaneous application of medium sized areas (4 m^2^), quite sharp peaks occur in the near-field concentration curves, there is a broadening effect if large areas are applied continuously and a moving worker is assumed. As in the well-mixed room case, the release of H_2_O_2_ is delayed at the beginning, while glutaraldehyde starts to evaporate earlier to some extent. It has to be noted that scenarios № 4 and 6 are quite similar with regard to the shape of the concentration time curves. This is because the worker, and with him the near field, moves and hence a significant portion of the applied area then contributes to the emission into the far field, where the air concentrations are rising accordingly.

A similar picture is obtained if there are two application cycles that are separated by a non-application break (s. black curves in Table 4, scenario № 7). Since the worker is assumed to move, a significant portion of the applied area contributes to the emission into the far field after a while, resulting in a rise in air concentrations. In contrast to scenario 6, however, there are now two peaks in air concentration that are particularly pronounced for the lower volatile substances. The delayed occurrence of the concentration peaks of H_2_O_2_ and glutaraldehyde suggests that the worker should leave the room at least after the application phase to minimize exposure.

It has to be noted that all simulation results presented in Table 4 were calculated using *N*_t_ *=* 10,000 iteration steps (Δ*t* = 1.8 s), both for Runge–Kutta and Euler, which has proven to be a good compromise between computing speed and accuracy.

**Figure 5:**
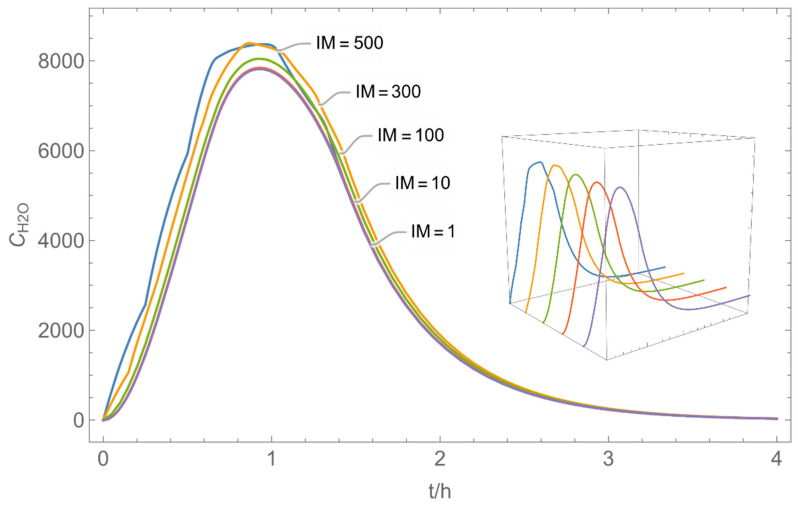
Concentration curves with different *IM* values for *N*_t_ = 10,000.

**Figure 6:**
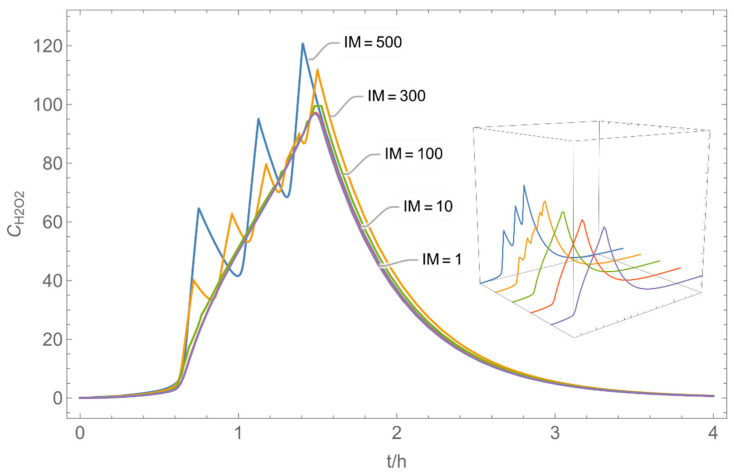
Concentration curves with different *IM* values for *N*_t_ = 10,000.

The integer multiple *IM* for Δ*t* were set to 1, that is the spatial resolution and the calculation time were maximal. In our work, we have also investigated to some extent how the temporal and spatial resolution determined by the size of *N*_t_ and *IM,* respectively, influences the numerical precision of this approach. In Figure 5 and Figure 6, the results of some example calculations for scenario 4 are depicted. The concentration curves of H_2_O and H_2_O_2_ reveal that *IM* values of 10 do not significantly change the shape of the curves. At the same time; however, the computing time, which is in the range of minutes for *IM = 1*, is reduced by a factor of about 1/10 (1/*IM*). Even with *IM* values of 100, the calculation accuracy may be acceptable for a quick rough estimate, with calculation times in the range of seconds.

## 4. Discussion

A key learning from the example calculations is that the proposed iterative algorithm is quite flexible, covers a wide range of scenarios and allows for variable application pattern in well-mixed room and near-field/far-field model configurations. Exposure assessors have traditionally used the concept of the well-mixed room together with constant emission rates of contaminants to predict the air concentrations at workplaces. The new algorithm is able to model time-dependent emission rates which are driven by the evaporation of mixtures applied to small and large surfaces. The example calculations have shown that backpressure effects should be considered in any case if evaporation from large surfaces takes place, because high backpressures can influence the time course of airborne concentrations by significantly delaying substance release. This applies to the well-mixed room and the near-field/far-field model as well. It may be intuitively clear that exposure in the near-field is higher than in the far field, which is clearly confirmed by the example calculations if the product is applied instantaneously to a small/medium sized area located inside the near-field (s. № 5). In other words, if products are applied to small/medium surfaces in large rooms, the near-field/far-field concept should be used to avoid underestimation of exposure.

At the same time, the example calculations reveal that continuous application at real workplaces should not be modelled with the near-field/far-field concept assuming instantaneous application because then the models tend to significantly overestimate peak exposure (s. № 1 and 5).

However, the situation changes somewhat if continuous (intermittent) application to large surfaces prevails, where the model allows for a moving near field (s. № 6 and 7). As the worker and with him the near field moves, a significant portion of the applied area can contribute to the emission into the far field after a while, resulting in comparable air concentrations in the near- and in the far field. Perhaps these results are intuitively obvious as well, but they can now be better supported quantitatively by the model.

Although the example calculations are restricted to aqueous binary mixtures, the proposed approach can be used for other multicomponent mixtures as well. Special attention should be paid to mixtures whose components differ greatly in polarity. In these cases, the evaporation of components from mixtures can deviate substantially from ideal behaviour, possibly leading to delayed or advanced release. On the other hand, the non-ideal effect is often less pronounced, and it may be argued that non-ideal solution calculations are not necessary for certain mixtures. In practice, however, only people with in-depth thermodynamic knowledge can predict when mixtures do not deviate significantly from Raoult’s law. In addition, there is often a lack of measured interaction parameters, which are needed by activity prediction models such as UNIFAC, AIOMFAC etc. Hence, it is recommended to consider the relevance of non-ideal behaviour prior to any assessment. At the same time, exposure and risk assessors should be aware of backpressure effects and deviations from ideal behaviour because the delayed or premature release of hazardous substances can determine the risks workers are facing and necessary risk management measures (e.g., restricted access) significantly.

Although the scenarios discussed above were aimed at reflecting potentially possible scenarios occurring at real workplaces, it has to be noted that scenario parameters are a bit arbitrarily chosen; hence, other options are possible. This applies to product concentrations, sizes of application areas, work rates and air exchange rates and “geometry”-related parameters as the volumes of the near and far field and their corresponding dimensions. However, as a matter of course, the model algorithm allows for any other combination of model parameters lying within the model boundaries as well. A more comprehensive evaluation of the model features including multicomponent mixtures is therefore planned for future research. In addition, model parameters are always subject to aleatoric and epistemic uncertainties [37,38]. That is, model parameters (e.g., work rate, airflows etc.) are inevitably variable at real workplaces and there is often a lack of knowledge (about the real process), respectively. In other words, aleatoric uncertainty refers to the irreducible part of the (total) uncertainty, whereas epistemic uncertainty refers to the reducible part. Although epistemic uncertainty can be diminished, there is always a trade-off between predictive power of a model and the needed model complexity. For instance, we decided not to go beyond the near-field/far-field approach because validation studies reveal a reasonable agreement with experimental data [39,40,41,42,43,44,45], whereas more sophisticated models such as CFD require a substantially higher amount of expenditure.

This paper predominantly outlines the theoretical concept of the new approach; hence, validation of the whole concept with measured data is still missing. However, as indicated above, there is some evidence in published literature that simple well-mixed room and near-field/far-field assumptions about airflow and contaminant transport patterns often lead to reasonable exposure estimates. Regarding the evaporation model we used for the development of the new approach, it has to be noted that Gmehling and Weidlich [9,14] compared exposure estimates with workplace measurements and carried out wind tunnel experiments for different solvents released from mixtures at low air velocities. Hence, this validation exercise can best represent the laminar flow conditions. If mass transfer is governed by fast turbulent air flows, which may lead to turbulent back mixing above the evaporation surface, other models may be more appropriate [12]. Furthermore, the influence of chemical reactions on the concentration course is not yet taken into account in the current version but can play a significant role in practice (e.g., reaction of H_2_O_2_ with biofilms). Although these effects can in principle be considered in the mass balance to some extent, the corresponding kinetics are regarded as complex and hence as a challenge for future research. Finally, it has to be noted that the proposed “well-mixed room” one- and two-box models are currently limited to scenarios where technical control measures such as local exhaust ventilation or containments are not used. However, as Ganser and Hewett [46,47] demonstrated, one- and two-box models can be extended to situations where various forms of a local control with exhaust are involved. This can be a topic for future work.

## 5. Conclusions

A method for predicting inhalation exposure resulting from the evaporation of volatile multicomponent mixtures has been developed. Based on an extended Euler algorithm, using time and space discretisation, numerical solutions of the underlying differential equations can be obtained for a wide variety of exposure scenarios, making this approach very flexible. Since the variety of evaporation settings in terms of geometry and air flow is endless, both one-box and near-field/far-field approaches are included to reflect spatial variability that can play a role, especially in larger rooms. Results can also be obtained for different application pattern, including (idealized) instantaneous application to surfaces and more practice-related continuous or intermittent spreading of products. In addition, it is possible to model situations in which the worker and the corresponding near field are stationary, and even situations in which the worker and the corresponding near field are moving in larger rooms.

Basically, time-varying predictions can be obtained for an arbitrary number of mixture components, including liquid-phase non-idealities as expressed by activity coefficients. The activity coefficients can be determined by experimental data or estimated by group contribution methods (e.g., UNIFAC). Although non-ideal behaviour is often less pronounced in practice, mixtures whose components differ greatly in polarity should be paid special attention. At the same time, the results of the example calculations suggest that even moderate deviations from ideality can lead to delayed or advanced release of components, possibly requiring adopted risk management measures.

Risk assessors should also be aware of backpressure effects. The example results suggest that situations where high evaporation rates from large surfaces occur can influence the time course of airborne concentrations by significantly delaying substance release. This applies to the one-box and the near-/far-field model as well.

Finally, it has to be noted that although the example calculations are restricted to aqueous binary mixtures, the proposed approach can be used for multicomponent mixtures as long as backpressure effects and deviations from ideal solutions are addressed adequately.

## Figures and Tables

**Table 1:** Definition of model parameters.

Indices:	
_i_	counting index for particular substances
_l_	counting index for particular area elements
_k_	counting index for particular time steps
_m_	counting index for the application cycle

_room_	parameters referring to the air space in the room in case of well-mixed-room models
_nf_	parameters referring to the air space in the near field in case of two-box models
_ff_	parameters referring to the air space in the far field in case of two-box models
_nf/ff_	used to indicate an air exchange rate between near and far field
_vent_	parameters referring the air exchanged by ventilation
_liq_	parameters referring to the liquid layer
_air_	parameters referring to air
_evap_	parameters indicating an evaporating fraction
_A_	parameters referring to the entire treated surface
_ΔA_	parameters referring to a fraction of the treated area (“area element”)
_app_	refers to the application duration
_expo_	refers to the total simulated exposure duration
_init_	initial value (for product amounts applied or air concentrations)
_a_	refers to starting time of application cycle
_b_	refers to end time of application cycle
_P_	refers to the entire product

*Parameters and units:*	
*p*:	vapour pressure [Pa]
p^*^:	vapour pressure of a pure substance [Pa]
*x:*	molar fraction
*γ*	activity coefficient
A:	surface area of the applied product [m^2^]
𝛽:	mass transfer coefficient [m/s]
R:	ideal gas constant (8.3145 Pa m^3^ K^−1^ mol^−1^)
T:	temperature [K]
V:	volume of an air space [m^3^]
$v_{\;}:$	velocity [m/s]
D:	molecular diffusion coefficient in air [m^2^/s]
$\nu^{\;}:$	kinematic viscosity [m^2^/s]
M :	molecular weight [kg/mol]
*n*:	molar amount [mol]
*w*:	weight fraction
*SC:*	the initial surface coverage with the product or a compound [kg/m^2^]
*C*:	concentration [kg/m^3^]
*t*:	time [s]
*Q*:	air exchange rate [m^3^/s]
*ACPH*:	number of air changes [1/h]
*r*:	work rate (rate at which the surface is covered with product) [m^2^/s]
*N*_t_:	number of time steps
*N*_ΔA_:	number of area elements
*IM*:	integer multiples of Δ*t*
*N*_AC_:	number of application cycles
*h*_A_:	height of the area to which the product is applied

**Table 2:** Constant input parameters.

*T =* 298.15 K	ν_air_ = 1.53∙10^−5^ m^2^/s	*V*_room_ = 200 m^3^
p*_H2O_ = 3130 Pa	*β*_H2O_ = 2.4∙10^−3^ m/s	*V*_nf_ = 8 m^3^
p*_H2O2_ = 257 Pa	*β*_H2O2_ = 2.2∙10^−3^ m/s	*Q*_nf/ff_ = 600 m^3^/h
p*_glutarald_. = 62 Pa	*β*_glutarald_. = 1.9∙10^−3^ m/s	*ACPH* = 2 /h
*D*_H2O_ = 2.4∙10^−5^ m^2^/s	*M*_H2O_ = 18∙10^−3^ kg/mol	*r* = 1 m^2^/min
*D*_H2O2_ = 1.8∙10^−5^ m^2^/s	*M*_H2O2_ = 34∙10^−3^ kg/mol	*ν*_air_ = 0.5 m/s
*D*_glutarald_. = 0.73∙10^−5^ m^2^/s	*M*_glutarald_. = 100∙10^−3^ kg/mol	*SC*_P_ = 0.1 kg/m^2^
*η* = 1.82∙10^−5^ kg/(m∙s)	*w*_i_ = 0.01	$C_{i,\mathrm{init}}$ = 0 mg/m^3^

**Table 3:** Variable model input for the example scenarios.

№	Model/ Algorithm	Area A [m^2^]	Application Time/Pattern	Back-Pressure	Activity Coeff.
1	WMR_inst/ Runge-Kutta	4	instantaneous	with and without	with
2	WMR_inst/ Runge-Kutta	40	instantaneous	with and without	with
3	WMR_inst Runge-Kutta	40	instantaneous	with	with and without
4	WMR_kont/ Euler	40	continuous over 0.67 h (40 min)	with	with and without
5	NF/FF_inst/ Runge-Kutta	4	instantaneous	with	with
6	NF/FF_mov/ Euler	40	continuous over 0.67 h (40 min)	with	with
7	NF/FF_mov_int/ Euler	40	intermittent: *t*_a,1_ = 0 h, *t*_b,1_ = 0.25 h, *t*_a,2_ = 0.42 h, *t*_b,2_ = 0.67 h	with	with

**Table 4:** Airborne concentration in mg/m^3^ vs. time in h for the various scenarios.

№	H_2_O/H_2_O_2_	H_2_O/Glutaraldehyde
1	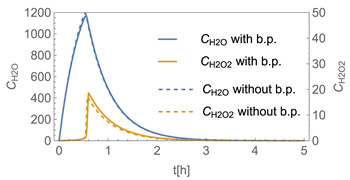	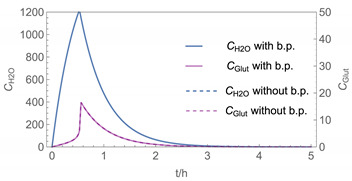
2	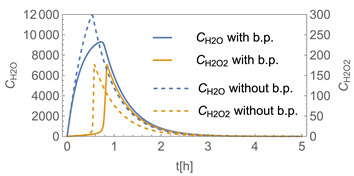	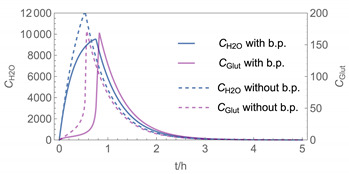
3	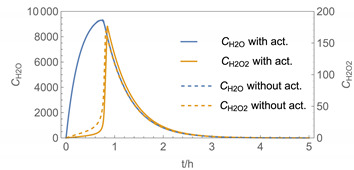	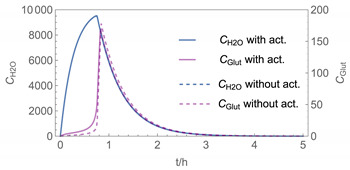
4	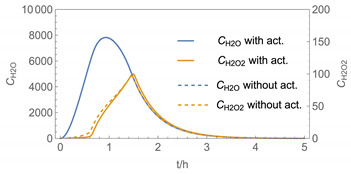	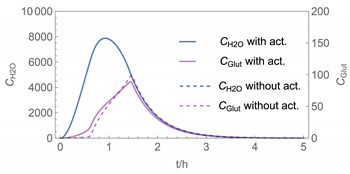
5	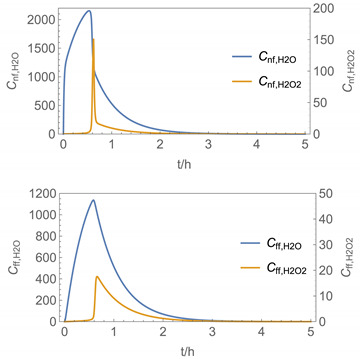	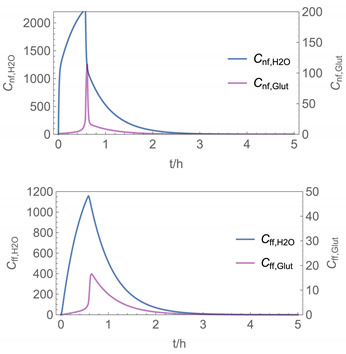
6	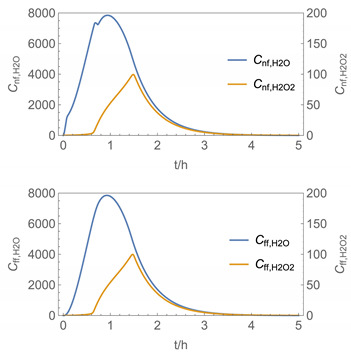	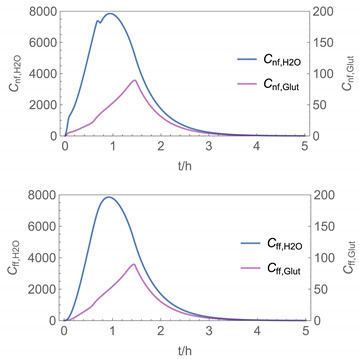
7	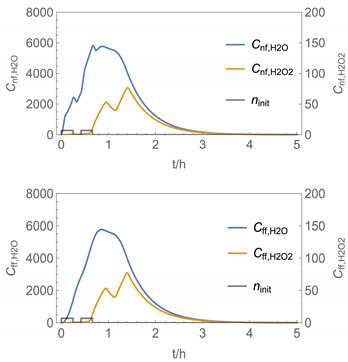	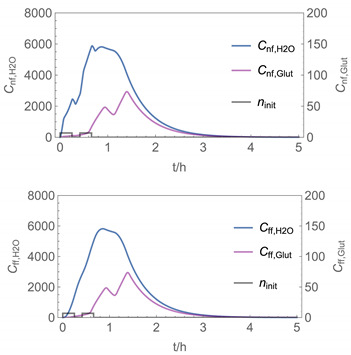

## Appendix A

### Appendix A.1. Pseudocode for the WMR_cont Scenario Using the Extended Euler Method

This piece of pseudocode, which is based on Mathematica, is only for clarification of the basic ideas and is restricted to a binary mixture. The algorithm starts with the definition of input parameters and corresponding functions and relations. The functions $f_{1}$ , $f_{2}$ , $h_{1}$ , $h_{2}$ are defined according to Equations (14) and (16) and the sums $g_{1}$ , $g_{2}$ according to Equation (15), respectively. The functions for the activity coefficient $\gamma_{1};\;\gamma_{2}$ must be specified depending on the corresponding substances. For intermittent application, the initial values $n_{\mathrm{liq},l,I,\mathrm{init}}$ for each new area element added are calculated with the initial surface coverage $SC_{i,m}$ , the coverage velocity *r* and the starting time *t*_a,m_ and end time *t*_b,m_ of an application cycle m according to the function *q*_i_ (for abbreviation), defined by Equation (20). The initial values for the airborne concentrations are $C_{1}\left(0\right)$ *,* $C_{2}\left(0\right)$ (that is, *t*_k_ = 0) and must be defined as well.

Discretizing the involved variables is achieved using Mathematica lists. Please note that the indices k and l are depicted in square brackets. The iteration algorithm is implemented by two nested Do loops. The k-loop represents the time discretization and the l-loop the area discretization taking into account the integer multiples *IM* of Δ*t*. Finally, the airborne concentration *C*_i_ (*t*) as a discrete function time *t* is obtain by transposing the *t*_k_ and *C*_i_ (*t*_k_) lists.

### Appendix A.2. Pseudocode for Continuous Application of a Binary Mixture in a Moving Near-Field Scenario Using Euler’s Method

In analogy to the well-mixed room case the pseudocode for a moving near-field scenario implements the iteration algorithm by two nested for next loops. For simplification, IM is set to 1. The outer k loop represents the time discretization and the inner l loop the area discretization. The iteration process over time starts with initial value $n_{\mathrm{liq},\mathrm{nf},i,\mathrm{init}}$ for each new area element added (that is k = l). For intermittent application, the initial values can be calculated using the function defined by Equation (20), which is for abbreviation denoted as *q*_i_. $n_{\mathrm{liq},\mathrm{ff},i,\mathrm{init}}$ is zero because no product i is initially applied to the far field. However, when the worker starts moving, this turns more and more applied near-field areas into applied far-field areas that can act as a far-field emission source. Whether a near-field area element starts emitting to the far field is decided by the criteria $N_{\Delta A,\mathrm{nf}}=k-l$ . The initial value for the airborne concentration of component i is $C_{\mathrm{nf},i}$ and $C_{\mathrm{ff},i}$ (that, is k = 1), which are generally assumed to be zero. The functions *fN*_i_, *fF*_i_, *hN*_i,_ *hF*_i,_ and the sums *gN*_i_, *gF*_i_ are defined according to the Equations (20), (21), (27) and (28).
