# Correction: Tischer, M.; Roitzsch, M. Estimating Inhalation Exposure Resulting from Evaporation of Volatile Multicomponent Mixtures Using Different Modelling Approaches. *Int. J. Environ. Res. Public Health* 2022, *19*, 1957

**DOI:** 10.3390/ijerph19169834

**Published:** 2022-08-10

**Authors:** Martin Tischer, Michael Roitzsch

**Affiliations:** BAuA: Federal Institute for Occupational Safety and Health, Unit “Exposure Scenarios”, Friedrich-Henkel-Weg 1-25, 44149 Dortmund, Germany

There was an error in the original publication [1]. In the last term of the right-hand side of Equation (8), the denominator *V_room_* is missing. Therefore, for the academic rigor of the paper, we modify Equation (8) to the correct form. The corrected Equation (8) appears below.

(8) $$\frac{dC_{\mathrm{room},i}}{dt}=\frac{M_{i}}{V_{\mathrm{room}}}\cdot \frac{dn_{\mathrm{evap},i}}{dt}-\frac{Q_{\mathrm{vent}}}{V_{\mathrm{room}}}\cdot C_{\mathrm{room},i}+\frac{Q_{\mathrm{vent}}}{V_{\mathrm{room}}}\cdot C_{\mathrm{vent},i}$$

The authors apologize for any inconvenience caused and state that the scientific conclusions are unaffected. The original publication has also been updated.
